# Evaluation of Molecular Methods for the Detection and Quantification of Pathogen-Derived Nucleic Acids in Sediment

**DOI:** 10.3389/fmicb.2017.00053

**Published:** 2017-01-24

**Authors:** Kata Farkas, Francis Hassard, James E. McDonald, Shelagh K. Malham, Davey L. Jones

**Affiliations:** ^1^School of Environment, Natural Resources and Geography, Bangor UniversityBangor, Wales; ^2^School of Ocean Sciences, Bangor UniversityBangor, Wales; ^3^School of Biological Sciences, Bangor UniversityBangor, Wales

**Keywords:** dPCR, norovirus, *Vibrio*, nucleic acid extraction, pathogen detection, qRT-PCR, sediment

## Abstract

The accurate detection of pathogens in environmental matrices, such as sediment, is critical in understanding pathogen fate and behavior in the environment. In this study, we assessed the usefulness of methods for the detection and quantification of *Vibrio* spp. and norovirus (NoV) nucleic acids in sediment. For bacteria, a commonly used direct method using hexadecyltrimethylammonium bromide (CTAB) and phenol-chloroform-isoamyl alcohol (PCI) extraction was optimized, whereas for NoV, direct and indirect (virus elution—concentration) methods were evaluated. For quantification, commercially available quantitative PCR (qPCR) and reverse transcription qPCR (RT-qPCR) kits were tested alongside a digital PCR (dPCR) approach. CTAB-based extraction combined with 16 h polyethylene glycol 6000 (PEG6000) precipitation was found to be suitable for the direct extraction of high abundance bacterial and viral nucleic acids. For the indirect extraction of viral RNA, beef extract-based elution followed by PEG6000 precipitation and extraction using the NucliSENS® MiniMag® Nucleic Acid Purification System and the PowerViral® Environmental RNA/DNA Isolation Kit and qRT-PCR resulted in 83–112 and 63–69% recoveries of NoV, respectively. dPCR resulted in lower viral recoveries (47 and 9%) and ~4 orders of magnitude lower *Vibrio* concentrations (3.6–4.6 log_10_ gc/100 g sediment) than was observed using qPCR. The use of internal controls during viral quantification revealed that the RT step was more affected by inhibitors than the amplification. The methods described here are suitable for the enumeration of viral and/or bacterial pathogens in sediment, however the use of internal controls to assess efficiency is recommended.

## Introduction

Pathogenic bacteria and viruses, found in environmental water due to wastewater discharge, agricultural activities, and run-off have been shown to associate with waterborne outbreaks of human disease (Radin, 2014). Pathogens in water readily adsorb to both inorganic and organic matter (Jin and Flury, 2002) resulting in the accumulation of pathogens in sediment (Staley et al., 2012). Hence, viruses and bacteria are often found in surface sediment in significantly higher concentrations than in the overlying water column (Rao et al., 1986; Duhamel and Jacquet, 2006; Vignaroli et al., 2013; Perkins et al., 2014). Furthermore, the association of viral and bacterial particles with sediment particles has been shown to increase the persistence of those pathogens (Gerba and McLeod, 1976; Smith et al., 1978; LaBelle and Gerba, 1980; Davies et al., 1995; Anderson et al., 2005). Pathogens may be released from sediment to water as a result of physical disturbance or variations in physico-chemical properties of water due to weather changes (An et al., 2002; Haramoto et al., 2009) and can be ingested by crustacea and shellfish destined for human consumption (Landry et al., 1983; Oliveira et al., 2011; Lowther et al., 2012). These events can result in public health threats far from the source of contamination.

In order to assess the potential health risks related to the contamination of the sediment, the development of reliable methods for the detection and accurate quantification of pathogens is essential. Bacterial contamination of effluent discharges and bathing water quality is traditionally measured by culture-dependent microbiological plating (International Organization for Standardization, ISO 9308-1:2014, 2014). Even though bacterial culturing is a simple and inexpensive approach, recent studies suggest that culture-dependent methods under-represent the bacterial abundance, as viable but non-culturable (VBNC) bacteria cannot be reliably enumerated in this way (Pinto et al., 2015). Pathogenic viruses have also been studied in environmental matrices using cell or tissue culturing, however those methods are time-consuming, require experienced staff and the results often underestimate viral titer due to the aggregation of viral particles (Charles et al., 2009; Farkas et al., 2015). Furthermore, many pathogenic viruses (e.g., human noroviruses) cannot be propagated *in vitro*.

Molecular approaches such as quantitative (qPCR) and reverse transcription qPCR (RT-qPCR) are often used for the rapid and accurate enumeration of pathogen-derived nucleic acids in environmental samples (Dick and Field, 2004; Bae and Wuertz, 2009; Furet et al., 2009; Girones et al., 2010) including sediment (Green and Lewis, 1999; Miura et al., 2011; Kim et al., 2014; Staggemeier et al., 2015a). PCR-based methods have shown the most promising results in terms of viral or bacterial recovery, however some chemical and biological compounds (e.g., nucleases, humic substances) within the sediment may inhibit the enzymes of RT and/or PCR resulting in false negative results (Meschke and Sobsey, 1998; Rock et al., 2010). However, progressive development of commercially available qPCR and qRT-PCR kits has resulted in procedures more resistant to inhibition.

Digital PCR (dPCR) using water-oil emulsion droplets or chip-based technology have been frequently used in diagnostics and virus detection (Sedlak and Jerome, 2013; Ding and Mu, 2016). Recently the usefulness of dPCR-based quantification for water and fruit samples has also been investigated (Ishii et al., 2014; Coudray-Meunier et al., 2015; Fraisse et al., 2017). Results have suggested that dPCR allowed a more accurate quantification of viral nucleic acids than q(RT-)PCR and was not adversely affected by high concentrations of humic acid (Hoshino and Inagaki, 2012) and other inhibitors associated with environmental samples (Fraisse et al., 2017). Although molecular approaches are unable to address the infectivity of the target pathogen, these methods have been widely used, especially for those viruses that cannot be cultured *in vitro*.

Approaches commonly used for the extraction of pathogens and pathogen-derived nucleic acids from sediments have shown high variability in efficiency (Miura et al., 2011), particularly among different types of sediment (Johnson et al., 1984). Comparison between studies is often challenging due to the various extraction and quantification approaches used (Rames et al., 2016). To facilitate detailed studies on viruses and bacteria, further validation of the protocols currently used for quantification is necessary. The aim of this study was to assess the efficiency of different extraction and PCR-based quantification methods for the recovery of bacteria (*Vibrio* spp.) and NoV commonly found in coastal and marine environments. Bacterial DNA was extracted directly from sediment. For virus recovery, direct and traditional, indirect elution—concentration methods were used. *Vibrio* spp. and NoV nucleic acids were then quantified using q(RT-)PCR and dPCR-based approaches.

## Materials and methods

### Environmental sediment samples

**Figure 1:**
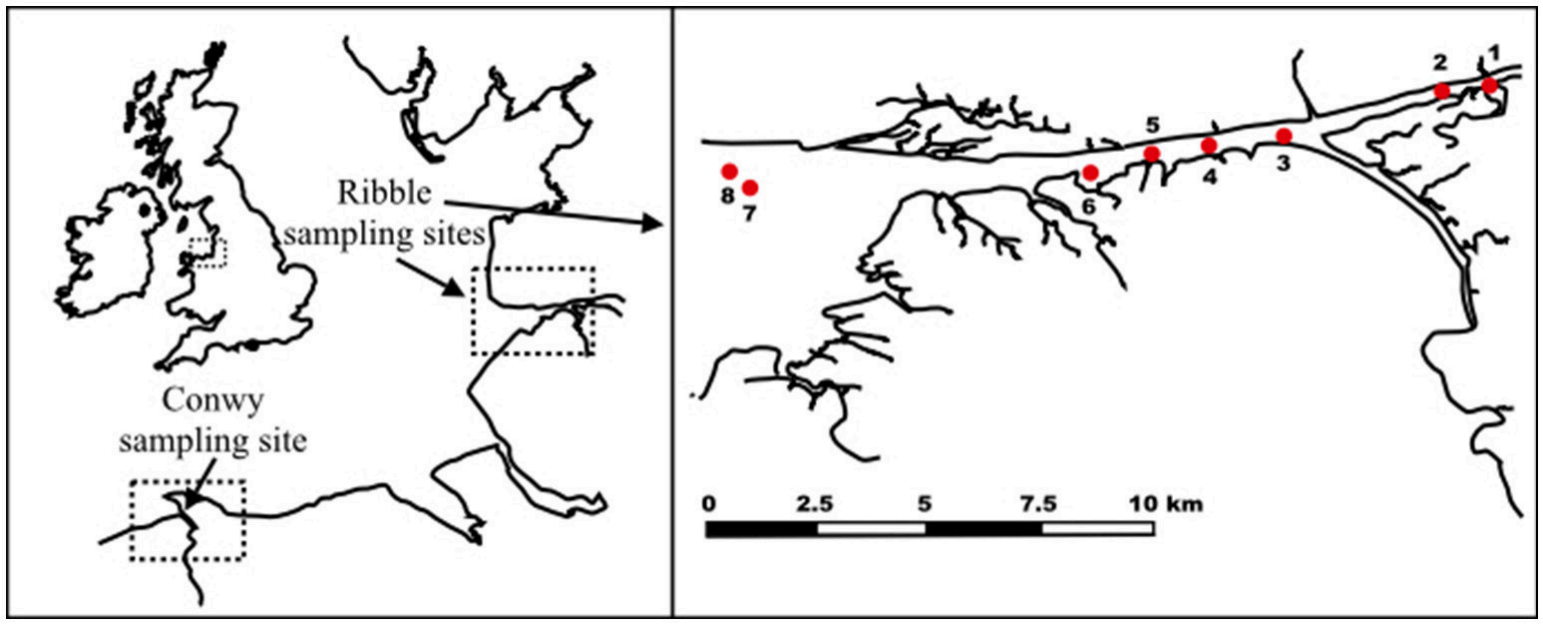
**Sampling sites at River Conwy and estuary (North Wales, UK) and River Ribble and estuary (North England, UK)**.

For the bacterial enumeration experiments, sediment samples from eight different sites (Site 1: 53°44′43.50″N, 2°49′14.30″W, Site 2:53°44′36.80″N, 2°49′49.10″W, Site 3: 53°44′7.48″N, 2°51′43.29″W, Site4: 53°44′3.70″N, 2°52′39.10″W, Site 5: 53°43′58.50″N, 2°53′20.90″W, Site 6: 53°43′55.50″N, 2°54′6.10″W, Site 7: 53°43′43.90″N, 2°58′14.00″W, Site 8: 53°43′54.40″N, 2°58′29.60″W) were collected from the River Ribble and estuary, North England, UK at high tide (Figure 1). The top 10 cm of sediment was collected by a mechanically operated Van Veen grab, aseptically placed in a 50 mL polypropylene centrifuge tube and stored at 4°C. Subsequently, small aliquots (~1 g subsamples in triplicates) from each replicate grab were frozen at −80°C pending nucleic acid extraction.

For the NoV experiments, sediment samples were collected in the Conwy estuary (53°16′N 3°49′W), North Wales, UK during receding tide. The top 1 cm layer of sediment was collected manually in sterile plastic bottles and stored at 4°C. Preliminary findings suggested that the sediment samples contained no NoV, thus those were subject to virus spiking. Prior to seeding with viruses, sediment was aliquoted (5, 2, and 0.25 g/tube) and autoclaved in order to inactivate bacterial enzymes which could interfere with the spiking studies.

### Norovirus seeding

Human norovirus (kindly provided by Prof. Ian Goodfellow, University of Cambridge, UK) was isolated from an anonymized clinical sample, collected as part of an ethically approved study at the University of Cambridge lead by Dr. Lydia Drumwright. The viral sample was generated by the preparation of a 10% solution using phosphate-buffered saline, pH 7.4 which was subsequently filtered through a 0.2 μm filter. An RNase treatment was performed on 100 μL 10-time diluted sample as described in Topping et al. (2009). The test revealed no significant concentration loss compared to a non-treated sample, suggesting that the sample contains predominantly intact NoV particles. NoV was added to sterilized sediment to achieve a final concentration of ~2 × 10^5^ genome copies (gc)/g sediment. Experiments were set up in triplicates and with one negative control (no virus added). Samples were incubated at room temperature on an orbital shaker at 90 rpm for 30 min to allow the attachment of viral particles to sediment.

### Extraction of bacterial DNA

Bacterial genomic DNA (gDNA) was extracted from 0.5 g sediment in triplicates using the direct extraction method based on the method of Griffiths et al. (2000). In brief, 0.5 mL glass beads, 0.5 mL of hexadecyltrimethylammonium bromide (CTAB) extraction buffer and 0.5 mL of phenol-chloroform-isoamyl alcohol (25:24:1; pH 8.0; PCI) were added to samples which were then lysed at 5.5 m/s for 30 s. Samples were centrifuged at 14,000 × g for 5 min and the top aqueous phase (containing nucleic acids) was transferred to a new tube. An equal volume of chloroform-isoamyl alcohol (24:1) (CI) was added, followed by centrifugation at 14,000 × g for 5 min. Nucleic acids were precipitated from aqueous layer by the addition of 2 volumes of 30% (wt/vol) polyethylene glycol 6000 (PEG6000)—1.6 M NaCl. The mixture was incubated at room temperature for 1, 2, or 16 h at 4°C and centrifuged at 16,000 × g for 10 min. The pellet was washed with 0.2 mL ice cold 70% ethanol and air dried prior to elution in 50 μL molecular-grade water with no subsequent pre-treatments. The concentration and quality of gDNA was checked using a Qubit fluorimeter 2.0 (Invitrogen, UK) and a Nanodrop ND-1000 (Nanodrop, USA), respectively. Results suggested high dsDNA concentrations in all samples, hence all samples were analyzed using qPCR as described below.

### Direct extraction of NoV RNA

The spiked sediment samples (0.25 g each) were mixed with 0.25 mL PBS. Viral nucleic acids were extracted directly for sediment using the CTAB-method described above. For PEG precipitation, samples were incubated at room temperature for 2 or 16 h at 4°C.

The suitability of commercial kits was also evaluated. Nucleic acids from 2 g NoV-spiked sediment were extracted using the PowerSoil® Total RNA Isolation Kit (MO BIO Laboratories, USA) according to manufacturer's protocol and eluted in 50 μL molecular-grade water. The PowerViral® Environmental DNA/RNA Isolation Kit (MO BIO Laboratories, USA) aiming to coextract viral RNA and DNA was also used. For that purpose, the spiked samples (0.25 g each) were mixed with 0.25 mL PBS prior to extraction. Beta-mercaptoethanol and PCI was added to the lysis buffer as advised by the manufacturer. Viral RNA was eluted in 50 μL molecular-grade water. The characteristics of each approach are summarized in Table 1.

**Table 1:** **Characteristics of the extraction methods used for the direct and indirect extraction of viral nucleic acids**.

**Extraction**	**Company**	**Cost per sample**	**Extraction type**	**Sample size**	**Processing time**	**Final volume**	**Additional equipment**	**Harmful chemicals**
CTAB-based extraction	N/A	£1	Physical, PEG	0.25 g	45 min + 12–16 h incubation	25–100 μL	Beadbeater	PCI, CI
PowerSoil® Total RNA Isolation Kit	MoBio	£8.60	Physical, Column	2 g	3 h	50–100 μL	None	PCI
PowerViral Environmental DNA/RNA Isolation Kit	MoBio	£5	Column	0.2 mL	45 min	50–100 μL	None	BME^*^
NucliSENS® MiniMag® Nucleic Acid Purification System	Bio Mérieux SA	£2.80	Magnetic beads	0.5 mL	45 min	50–100 μL	MiniMag system^*^	None

* *Optional*.

### Indirect extraction of NoV RNA

NoV particles were eluted and concentrated as described in Lewis and Metcalf (1988). In brief, 5 g NoV-spiked sediment samples were mixed with 15 mL 3% beef extract in 2 M NaNO_3_ (pH 5.5) for 30 min and the solid matter was removed by centrifugation at 2500 × g, 10 min. The pH of the eluent was adjusted to 7.5 and incubated in 15% PEG6000 and 2% NaCl overnight at 4°C and centrifuged at 2500 × g for 80 min. The resulting pellet was subject to nucleic acid extraction, using the CTAB-based extraction (detailed above) and two commercial kits, the NucliSENS® MiniMag® Nucleic Acid Purification System (bioMérieux SA, France), and the PowerViral® Environmental RNA/DNA Isolation Kit (MO BIO Laboratories, USA). Nucleic acids were extracted according to manufacturer's protocol and eluted in 50 μL molecular biology grade water. Prior to extraction using the MiniMag® System, samples were incubated in 10 mg/mL proteinase K solution at 37°C for 60 min. No PCI was used when samples were extracted using the PowerViral® Kit. The characteristics of each approach are summarized in Table 1.

### Quantitative PCR and RT-PCR

All qPCR assays were carried out in a QuantStudio™ Flex 6 Real-Time PCR System (Applied Biosystems, USA). For quantification, dilution series of a plasmid DNA carrying the target sequence were used (Primerdesign Ltd, UK). For all samples the original and a 10-times (vol:vol) diluted extract were tested. A positive NoV RNA control—extracted from fecal matter using the PowerViral Kit and 1000 × diluted in molecular-grade water—was also added to qRT-PCR reactions. The characteristics of each approach are summarized in Table 2.

**Table 2:** **Characteristics of the quantification kits used in this study**.

	**Kit**	**Company**	**Cost per sample**	**RT**	**Polymerase**	**Duration**
One-step	RNA Ultrasense®	Invitrogen	£5.80	Superscript® III	Platinum® *Taq*	3 h 40 min
	Oasig	Primer design	£2	N/A	N/A	2 h
Two-step	Superscript® IV RT	Invitrogen	£5.30	Superscript® IV	–	20 min
	KAPA Force Probe qPCR	KAPA Biosystems	£1.20	–	KAPA3G	2 h
	QuantStudio™ 3D Digital PCR	Applied Biosystems	£8	–	N/A	2 h 45 min

For detection of bacterial (*Vibrio* spp.) DNA by qPCR, two commercially available TaqMan qPCR mixes were tested, namely the Oasig qPCR Master Mix (Primerdesign Ltd, UK) and the KAPA Force Probe qPCR mix (KAPA Biosystems, USA). The 20 μL reaction mixes contained 1 × qPCR mix, 1 μg bovine serum albumin (BSA), 1 μL Vibrio spp. Primer/Probe mix (Primerdesign Ltd, UK), and 4 μL sample or standard. Using the Oasig qPCR Master Mix, the initial denaturation was 2 min at 95°C followed by 50 cycles of amplification consisting of 95°C for 15 s and 60°C for 60 s. Using the KAPA Force Probe qPCR mix, the 5 min denaturation at 98°C was followed by 45 cycles of amplification consisting of 95°C for 15 s, 60°C for 60 s, and 65°C for 30 s.

For detection of NoV RNA, three RT-qPCR approaches were tested. A single-step TaqMan-based qRT-PCR assay was used according to the method described previously by Flannery et al. (2014) using RNA Ultrasense One-step qRT-PCR kit (Invitrogen, USA). The 20 μL qPCR reaction mix contained 1 × RNA Ultrasense Reaction Mix with 1 μL RNA Ultrasense Enzyme Mix, 10 pmol of the forward (ATG TTC AGR TGG ATG AGR TTC TCW GA), 20 pmol of the reverse (TCG ACG CCA TCT TCA TTC ACA) primers, 5 pmol of the probe (FAM-AGC ACG TGG GAG GGC GAT CG-TAMRA), 0.1 × ROX reference dye, 1 μg BSA and 4 μL of the sample/plasmid DNA. Following a 60 min step of reverse transcription at 55°C and a 5 min step of denaturation at 95°C, the 45 cycles of amplification consisted of 95°C for 15 s, 60°C for 60 s, and 65°C for 60 s.

A single-step TaqMan-based qRT-PCR assay using the same NoV standards, primers, and probe as described above with the Oasig OneStep qRT-PCR Master Mix (Primerdesign Ltd., UK) was also used, according to the manufacturer's instructions. The 20 μL reaction mix contained 4 μL sample/standard and 1 μg BSA. The reverse transcription was performed at 42°C for 10 min followed by a 2 min denaturation at 95°C and 50 cycles of amplification consisting of 95°C for 15 s and 60°C for 60 s.

The usefulness of two-step RT-qPCR for NoV RNA quantification was also investigated. For RT the Superscript IV (Invitrogen, USA) was used with oligo (dT)_18_ primers according to the manufacturer's instructions. The 20 μL reaction mix contained 4 μL RNA extract. Following RT, the cDNA was quantified by qPCR using the KAPA Force Probe qPCR mix (KAPA Biosystems, USA) and the standards, primers and probe for NoV as detailed above. The 20 μL reaction mix contained 4 μL sample/standard and 1 μg BSA. The 5 min denaturation at 98°C was followed by 45 cycles of amplification consisting of 95°C for 15 s, 60°C for 60 s, and 65°C for 30 s.

### Nucleic acid quantification using dPCR

Digital PCR was performed using the QuantStudio™ 3D Digital PCR System using sealed chip technology (Applied Biosystems, USA). The 14.5 μL PCR mixture contained 7.5 μL QuantStudio™ 3D Digital PCR Master Mix v2, 0.8 μL of Norovirus genogroup 2 or *Vibrio* spp. primer/probe mix (Primerdesign Ltd, UK) and 5 μL sample. The reaction mixture was loaded onto a QuantStudio™ 3D Digital PCR 20 K Chip using an automatic chip loader. Amplification was carried out using the following thermal cycling conditions: 96°C for 10 min, then 39 cycles of 60°C for 2 min and 96°C for 30 s followed by 60°C for 2 min. After amplification the chips were allowed to cool to room temperature and then read on the QuantStudio™ 3D Instrument. The results were further analyzed using the QuantStudio 3D AnalysisSuite™ Cloud Software.

### Internal control for NoV quantification

The level of inhibition for each extract type was estimated using plasmid DNA incorporating the target sequence of the NoV quantification assays described above. The plasmid DNA was added to each negative RNA extract to reach a concentration of ~10^3^ copies/μL.

### Statistical analysis

Paired *t*-tests were used to analyze the qPCR results, the data was found to have no significant outliers between the two related groups. Next a Shapiro-Wilk test of normality revealed approximately normal distributions between each paired observation. Finally, a Welch's *t*-test was undertaken on SPSS version 22 (IBM, USA).

## Results and discussion

### Comparison of PEG incubation times on quantification of bacterial DNA from sediment

**Figure 2:**
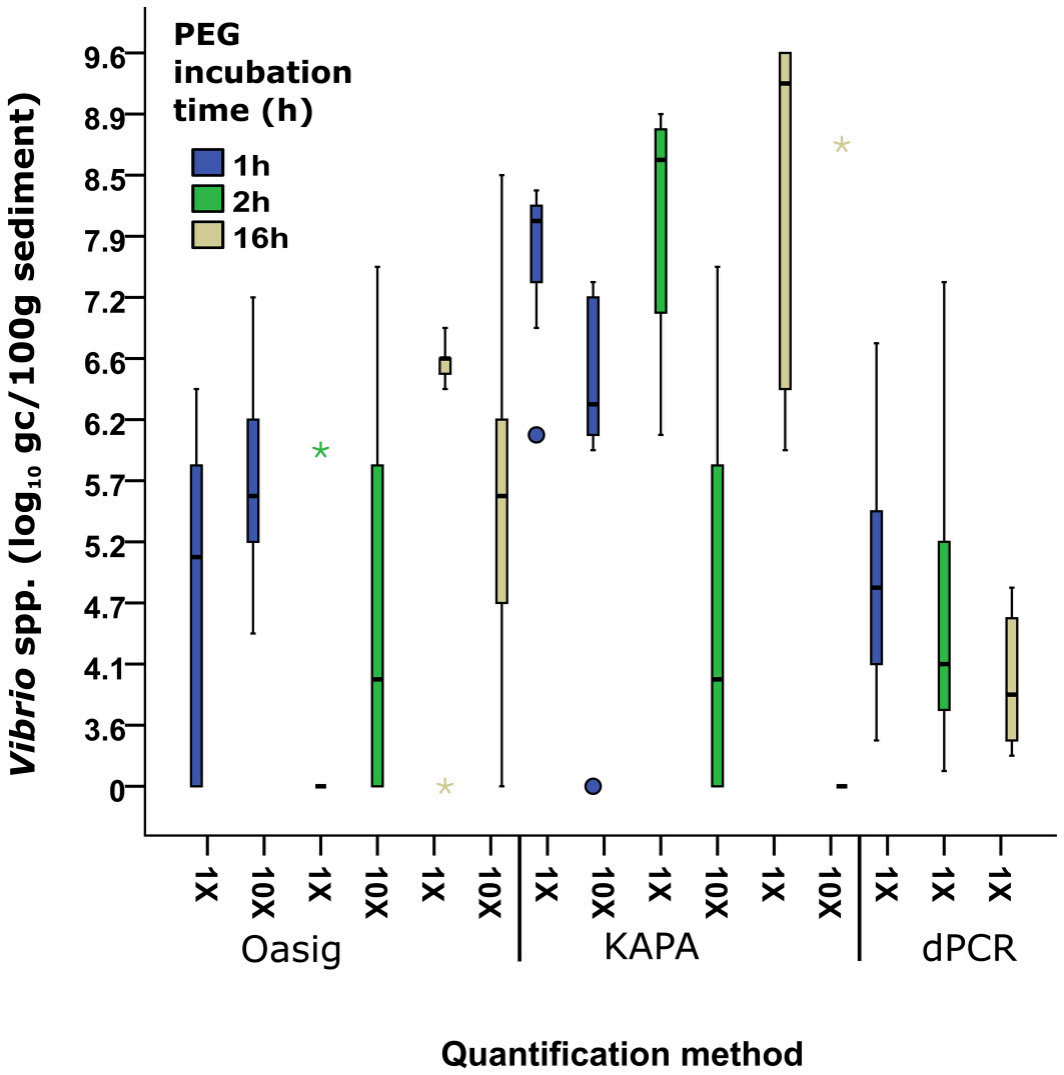
**Comparison between Oasig qPCR, KAPA Qpcr, and dPCR and PEG incubation time from samples extracted with Griffiths et al. (2000) on the quantification of ***Vibrio*** spp. from sediment**. Each bar represents pooled *Vibrio* spp. abundances from eight different sediment sample sites in the Ribble with three biological repeats at each site/treatment. Boxes represent 25–75% and median value (thick black line). Whiskers represent the minimum and maximum data. Outliers and extreme values are plotted as circles and stars, respectively.

The original protocol for direct extraction of bacterial nucleic acids from soil samples suggested the use of a 2 h incubation time to enable DNA binding to PEG (Griffiths et al., 2000) resulting in sufficient DNA precipitation. However, many other studies use longer PEG incubation times (Krsek and Wellington, 1999; Roose-Amsaleg et al., 2001). In order to investigate whether incubation times have an effect on bacterial DNA extraction efficiency, we incubated the samples in PEG solution for 1, 2, and 16 h. The results suggested that increased incubation times resulted in higher DNA concentrations when qPCR quantification was used (Figure 2). Between 1 and 16 h incubation time, the median *Vibrio* spp. abundance increased by 1.3 and 1.2 log_10_ gc/100 g sediment for the Oasig and KAPA qPCR method, respectively. In contrast, dPCR had a 0.7 and 0.1 log_10_ gc/100 g sediment reduction in *Vibrio* spp. abundance between 1–2 and 2–16 h of PEG incubation. This implies that prolonged PEG incubation resulted in inhibited quantification by dPCR. It has been suggested that PCR inhibitors can be concentrated along with nucleic acids during PEG precipitation (LaMontagne et al., 2002; Miura et al., 2011). Although, dPCR has been shown to be resistant to humic acid, a common inhibitor in environmental samples (Hoshino and Inagaki, 2012), our findings suggest that these sediment samples may have contained other organic substances that also interfere with dPCR amplification.

### Comparison of different methods for quantifying bacteria in sediment

*Vibrio* spp. quantification was significantly greater in all PEG incubation times with KAPA mastermix (range 7.9–9.2 log_10_ gc/100 g sediment) compared to Oasig mastermix (0–6.56 log_10_ gc/100 g sediment) and dPCR (3.6–4.6 log_10_ gc/100 g sediment; Figure 2). Overall the highest concentrations were achieved for the samples incubated in PEG solution overnight using the KAPA mastermix for quantification resulting in 1.7 × 10^9^ gc/100 g sediment on average. Dilution, 10 × (vol:vol) of DNA extracts did not improve the recovery suggesting limited inhibition using this mastermix. Significantly lower DNA concentrations or negative results were observed using the Oasig qPCR mastermix. Recovery of *Vibrio* spp. targets increased for the 1 and 2 h PEG treatments on diluted samples, suggesting inhibition of the mastermix. The observed average *Vibrio* spp. abundance increased by 0.5 log_10_ gc/100 g sediment for the 1 h PEG treatment (*p* < 0.05; average fold increase 1 × 10^6^ gc/100 g sediment). Dilution of the 2 h PEG samples allowed recovery of *Vibrio* spp. at three sites which gave a negative qPCR result using undiluted samples; this resulted in an average fold increase of 3 × 10^7^ gc/100 g sediment. However, the remaining five sites remained negative using qPCR result when testing on the diluted samples. In contrast, *Vibrio* spp. target recoveries decreased by 1 log_10_ gc/100 g sediment for the 16 h treatment with dilution, principally due to low initial nucleic acid concentration (typically <0.1 ng/μL). This highlights the role of PEG incubation on inhibitor carryover.

Interestingly very low *Vibrio* concentrations were observed using dPCR for quantification regardless of the PEG incubation times. It has been suggested that qPCR-based quantification may overestimate the actual DNA concentrations, whereas dPCR gives a more accurate estimate on DNA concentrations (Yang et al., 2014). However, the five orders of magnitude difference between the dPCR and qPCR results is higher than expected and may be due to the inhibition of the dPCR. Based on the high variation observed among samples analyzed by dPCR, the low concentrations may be due to analytical error.

The observed *Vibrio* concentrations using the KAPA mastermix correlated with previous findings on the concentration of *Vibrio* spp. in estuarine sediment. Using qPCR for quantification the *Vibrio* concentrations were 8–13 log10 gc/100 g estuarine sediment (Givens et al., 2014) and 4–9 log10 gc/100 g costal sediment (Vezzulli et al., 2009). Therefore, 16 h PEG incubation times when extracting nucleic acids and use of inhibitor resistant mastermix is recommended when quantifying bacteria/viruses from sediment samples.

### Direct extraction of viral RNA from sediment

The results of direct extractions of NoV RNA from sediment are summarized in Table 3. Typically, when samples were analyzed directly by qRT-PCR, very low, or no recovery was noted. However, when samples were diluted 10-fold prior to quantification, viral RNA could be recovered. As shown previously, PEG has the capacity to enrich inhibitors affecting the reverse transcriptase and DNA polymerase enzymes (Miura et al., 2011). Our results suggest that with sample dilution, the effect of inhibitors was eliminated and 82% recovery was achieved in samples incubated for 16 h in PEG solution. In contrast, NoV was not recovered with the original CTAB-based method of Griffiths et al. (2000) with 2 h PEG incubation regardless of sample dilution. In accordance with our findings on *Vibrio* spp. recovery (detailed above), these results suggest that the short incubation time did not allow for good levels of nucleic acid binding to PEG.

**Table 3:** **Genome copy numbers (gc) and recoveries (Rec%) of NoV RNA from spiked sediment samples using direct extraction methods**.

**Sample**	**Dilution**	**Concentration (gc/sample)**	***SD***	**Rec%**	***SD***
CTAB 16 h	Undiluted	0	0	0	0
CTAB 16 h	10 × diluted	1.2 × 10^5^	1.7 × 10^4^	82.1	11.4
CTAB 2 h	Undiluted	0	0	0	0
CTAB 2 h	10 × diluted	0	0	0	0
PowerSoil	Undiluted	4.5 × 10^1^	7.5 × 10^0^	<0.1	<0.1
PowerSoil	10 × diluted	3.0 × 10^4^	7.7 × 10^1^	20.5	0.1
PowerViral	Undiluted	3.5 × 10^1^	5.1 × 10^0^	<0.1	<0.1
PowerViral	10 × diluted	1.6 × 10^2^	6.3 × 10^1^	<0.1	<0.1

*Number of copies added to each sample: 1.47 × 10^5^. Diluted samples were ten times diluted prior to qRT-PCR. 2 and 16 h indicates the duration of PEG6000 incubation. SD represent standard deviations of independent experiments (n = 2)*.

The PowerSoil® Total RNA Isolation Kit also performed well for the direct recovery of viral RNA, however the recoveries were slightly lower than those achieved using the CTAB-based extraction with 16 h PEG incubation (21 vs. 82%). Even though the kit uses inhibitor removal technology and column-based purification after the PCI extraction, the dilution of the sample prior to qRT-PCR was necessary to accurately quantify viral RNA.

In contrast to the PowerSoil® and CTAB protocols, low recoveries (<0.1%) were observed in samples extracted using the PowerViral® Environmental DNA/RNA Isolation Kit. Although previous research has demonstrated successful recovery of viruses from environmental samples, such as biosolids and concentrated surface water, using this method (Iker et al., 2013), our results suggest that this approach is not adequate for sediment samples.

Direct extraction has been shown to be suitable for the detection of viral nucleic acids in environmental samples however, recoveries depend on the type of the sample and the target viruses (Miura et al., 2011; Honjo et al., 2012; Iker et al., 2013). The CTAB-based extraction has been widely used for studying microbial communities in soil (Amos et al., 2015; Mayer et al., 2015; Santos et al., 2015), and this study supports its usefulness for the recovery of high abundance viruses and bacteria from sediment. It is a cost-effective approach and requires less preparation time than the PowerSoil® Kit (Table 1). The major disadvantage of direct extraction is the use of hazardous chemicals and physical extraction in order to separate nucleic acids. These treatments may affect the integrity of the viral and bacterial genomes, although this is unlikely to impact qPCR-based quantification directly.

### Effect of beef extract-based elution on nucleic acid extraction and RT-qPCR

Beef extract-based solutions are routinely used for the elution of infectious viral particles from environmental samples, e.g., sediment and water (Pepper and Gerba, 2000; Ikner et al., 2012). However, the residual beef extract may interfere with enzymes used for molecular quantification (Iker et al., 2016). In order to determine beef extraction-related inhibition, 3% beef extract in 2 M NaNO_3_ pH 5.5 solution was spiked with a known concentration of NoV and concentrated using PEG solution. Viral nucleic acids were extracted from the PEG pellet and quantified using PCR-based approaches.

Results suggested that beef extract solution did not significantly inhibit the extraction and qRT-PCR (Table 4). Using the RNA Ultrasense One-step qRT-PCR system for quantification, results suggested no inhibition on quantification using the MiniMag® System or the PowerViral® Kit. Using these extraction approaches, the NoV RNA was fully recovered, suggesting that the residual beef extract and the PEG had no effect on the extraction or quantification. As the CTAB-based extraction gave the best recoveries for the direct extraction of viral nucleic acids from sediment, its usefulness for indirect extraction was investigated. Results showed only 1% recovery, and sample dilution prior to qRT-PCR did not improve recovery efficiency. This suggested that the extraction was inhibited by residual beef extract and therefore this approach was not used in subsequent indirect extractions.

**Table 4:** **Genome copy numbers (gc) and recoveries (Rec%) of NoV from 3% beef extract in 2 M NaNO_**3**_, pH 5.5—PEG precipitation, followed by RNA extraction: NucliSENS® MiniMag® Nucleic Acid Purification System (MM), the PowerViral™ Environmental RNA/DNA Isolation Kit (PV), and the CTAB-based method adapted from Griffiths et al. (2000; CTAB)**.

**Quantification**	**Extraction**	**Undiluted nucleic acid extract**				**Diluted nucleic acid extract**			
		**Control**		**Sample**		**Control**		**Sample**	
		**gc**	**Gc**	**Rec%**	***SD***	**gc**	**gc**	**Rec%**	***SD***
RNA Ultrasense	MM	2.3 × 10^5^	2.8 × 10^5^	123.9	15.9	2.2 × 10^5^	2.3 × 10^5^	100.9	28.5
One-step	PV	2.3 × 10^5^	2.8 × 10^5^	112.6	54.4	2.1 × 10^5^	3.3 × 10^5^	133.0	44.3
qRT-PCR	CTAB	6.6 × 10^4^	1.7 × 10^3^	0.9	1.5	2.3 × 10^5^	2.2 × 10^3^	1.1	2.0
Oasig	MM	4.5 × 10^2^	3.1 × 10^2^	–^*^	–^*^	4.7 × 10^2^	3.4 × 10^2^	–^*^	–^*^
One-step	PV	5.6 × 10^1^	5.3 × 10^1^	–^*^	–^*^	6.3 × 10^1^	1.3 × 10^2^	–^*^	–^*^
qRT-PCR	CTAB	5.9 × 10^4^	0	0	0	0	0	0	0
Superscript IV RT	MM	1.7 × 10^5^	3.5 × 10^4^	12.4	3.3	2.6 × 10^5^	2.8 × 10^5^	97.9	3.0
KAPA force	PV	1.6 × 10^4^	5.3 × 10^4^	20.7	2.7	2.5 × 10^4^	2.3 × 10^5^	89.1	3.3
Probe qPCR	CTAB	2.7 × 10^3^	2.7 × 10^1^	<0.1	0	2.5 × 10^3^	9.0 × 10^3^	3.9	1.8

*Undiluted and 10 times diluted nucleic acid extracts were subject to quantification. SD represent standard deviations of experiments (n = 3)*.

* *Recoveries were not calculated due to the low RNA concentration observed in the controls*.

In order to further investigate the inhibitory effect of beef extract on quantification, different qPCR-based methods were tested. The Oasig OneStep qRT-PCR system did not provide the accurate quantification of NoV RNA in diluted fecal matter and in the beef extract samples (Table 4). The concentrations detected in both sample types were 3–4 orders of magnitude lower than those detected using the RNA Ultrasense One-step qRT-PCR system, regardless of the extraction method used (Table 4). The controls and dilution also showed lower concentrations than expected suggesting that the qRT-PCR conditions were sub-optimal. Nonetheless, increased RT-step and extension times did not improve the results (data not shown). Therefore, the Oasig OneStep qRT-PCR system was not deemed appropriate for the sample types/conditions examined in this study.

### Indirect extraction of viral nucleic acids from sediment

The results of virus recovery from sediment spiked with NoV using beef extract elution followed by PEG precipitation are summarized in Table 5. The method was shown to extract 80–100% of rotavirus and hepatitis A virus from sediment (Lewis and Metcalf, 1988) and used to quantify enterovirus, rotavirus, and hepatitis A virus (Le Guyader et al., 1994) and adenovirus (Staggemeier et al., 2015b) in sediment.

**Table 5:** **Genome copy numbers (gc) and recoveries (Rec%) of NoV from sediment using elution—concentration followed by RNA extraction: NucliSENS® MiniMag® Nucleic Acid Purification System (MM) and the PowerViral™ Environmental RNA/DNA Isolation Kit (PV)**.

**Quantification**	**Extraction**	**Control**	**Undiluted nucleic acid extract**			**Diluted nucleic acid extract**			***n***
		**gc**	**gc**	**Rec%**	***SD***	**gc**	**Rec%**	***SD***	
RNA Ultrasense	MM	3.8 × 10^5^	2.7 × 10^5^	70.9	14.6	4.3 × 10^5^	112.0	1.5	3
One-step qRT-PCR	PV	2.8 × 10^5^	2.8 × 10^4^	10.1	13.6	1.7 × 10^5^	62.8	19.7	3
Superscript IV RT KAPA Force	MM	3.4 × 10^5^				2.9 × 10^5^	83.1	1.6	2
Probe qPCR	PV					2.4 × 10^5^	68.6	3.7	2
Superscript IV RT	MM	4.0 × 10^5^				1.9 × 10^5^	46.7	–	1
dPCR	PV					3.6 × 10^4^	8.8	12.3	2

*Undiluted and 10 times diluted nucleic acid extracts were subject to quantification. SD represent standard deviations of independent experiments*.

In this study, different extraction and quantification methods for the recovery of NoV RNA were compared. The best recoveries were achieved using the MiniMag® System followed by qRT-PCR using the RNA Ultrasense system, however sample dilution prior to quantification was necessary to achieve full NoV recovery. Using the MiniMag® System followed by two-step RT-qPCR also resulted in high recoveries (83%) together with the PowerViral® Kit extraction followed by one-step or two-step RT-qPCR (63–69%). Using the two-step quantification approach, 4 μL of the RNA extract was the subject of the RT step, resulting in a five-time diluted sample. No further dilution was necessary prior to qPCR.

The cDNA sample derived from RT was additionally quantified using dPCR. For samples extracted using the MiniMag® System, the recovery rate (47%) was comparable with qPCR-based quantification, whereas recoveries observed in samples extracted using the PowerViral® Kit were significantly lower and showed high variation (0.2–17.5%) suggesting this extraction approach was not appropriate for dPCR quantification from sediments.

Previous reports have also shown that the MiniMag® System is suitable for the extraction of nucleic acids of enteric viruses from concentrated water samples treated with beef extract solution (Rutjes et al., 2005; Baert et al., 2011; Sano et al., 2011). The system has also been used for viral nucleic acid extraction from meat, fruits and vegetables (Butot et al., 2007; Summa et al., 2012). Furthermore, a method using the MiniMag® System for the extraction of hepatitis A virus and NoV for the detection of enteric viruses in shellfish has attained accreditation by the International Organization for Standardization (ISO; Lees and CEN WG6 TAG4, 2010) suggesting that the system is suitable for the extraction of viral nucleic acids from difficult environmental samples. The PowerViral® Kit has also been shown to outperform other commercial extraction kits when viral nucleic acids were extracted from fecal matter, biosolids, and concentrated water samples with residual beef extract (Iker et al., 2013). The extractions can be performed within 45 min and the use of non-standard equipment and hazardous chemicals is not a requirement of successful extraction (Table 1).

### Assessment of inhibition

In order to understand the inhibitory effect of environmental matrices on PCR amplification, the negative controls of direct and indirect extractions were spiked with known concentrations of plasmid DNA incorporating the target sequence of NoV qRT-PCR. Results suggested that the inhibitors extracted along with nucleic acids had no or little effect on the polymerase enzymes of the four quantification types when indirect extraction was used combined with MiniMag® or PowerViral® Kit extraction (Table 6). However, the negative results suggested that those samples where direct extraction was used contained high concentrations of inhibitors affecting the polymerase activity of the Oasig OneStep qRT-PCR and the KAPA Force Probe qPCR systems. Nonetheless, DNA could be precisely quantified using the RNA Ultrasense and dPCR systems regardless of extraction type. Results imply that the observed inhibition described in previous sections had a significant effect on reverse transcriptase but little impact on the polymerase enzymes.

**Table 6:** **Copy numbers and recoveries (Rec%) of plasmid DNA from negative NoV RNA extracts of: indirect extraction of sediment using NucliSENS® MiniMag® Nucleic Acid Purification System (MM), indirect extraction using PowerViral™ Environmental RNA/DNA Isolation Kit (PV), and direct extraction using CTAB-based method adapted from Griffiths et al. (2000; CTAB)**.

**Quantification**	**Extraction**	**copies**	**Rec%**	***SD***
RNA Ultrasense	Control	3.1 × 10^3^		
one-step	MM	3.3 × 10^3^	108.4	14.4
qRT-PCR	PV	1.7 × 10^3^	56.5	17.0
	CTAB	2.1 × 10^3^	69.0	5.7
Oasig	Control	3.0 × 10^3^		
One-step	MM	2.6 × 10^3^	88.2	9.5
qRT-PCR	PV	2.9 × 10^3^	97.1	18.0
	CTAB	0	0	0
KAPA Force	Control	3.0 × 10^3^		
Probe qPCR	MM	2.8 × 10^3^	91.6	8.6
	PV	2.8 × 10^3^	93.7	3.2
	CTAB	0	0	0
dPCR	Control	2.0 × 10^3^		
	MM	2.0 × 10^3^	96.1	15.4
	PV	1.7 × 10^3^	85.7	7.8
	CTAB	2.8 × 10^3^	136.1	3.8

*SD represent standard deviations of independent experiments (n = 2)*.

### q(RT)-PCR vs. digital PCR

In this study, we aimed to evaluate the usefulness of 3D dPCR for the detection and quantification of viral and bacterial nucleic acids. However, while the plasmid spiking experiments suggested no significant inhibition from environmental samples (Table 6), the results of the *Vibrio* experiments suggested some obstruction may be due to the presence of inhibitors or analytical limitations of the technology (Figure 2). Furthermore, dPCR only gives accurate quantification in a narrow concentration range (~10^4^–10^1^ DNA copies/μL), whereas qPCR covers a much broader range (10^6^–10^1^ DNA copies/μL). Both approaches failed to give accurate quantification for low concentration samples (<30 copies/reaction), however using q(RT-)PCR, a clear amplification curve was observed suggesting that those samples were positive. On the contrary, dPCR results for those samples were either considered negative or results were ambiguous due to background noise. Previous studies have achieved more reliable results on environmental samples using dPCR than with qPCR (Hoshino and Inagaki, 2012; Coudray-Meunier et al., 2015), however the water-oil emulsion droplet technology used by the equipment validated in those studies may give different recoveries from 3D dPCR system. Overall, based on our results, further validation and method development is essential in order to routinely use 3D dPCR approaches in environmental studies.

### Molecular detection of pathogen-derived nucleic acids

Nucleic acid extraction followed by qPCR-based quantification is the most commonly used method for the rapid detection of pathogens in environmental samples, allowing prompt response in case of a public health threat. However, false negative results may occur as a result of PCR inhibition or poor efficiency of nucleic acid extraction (Miura et al., 2011; Iker et al., 2013; Staggemeier et al., 2015b). Hence, the methods used for the detection of pathogens should be thoroughly validated. Sediments have the ability to accumulate bacterial and viral pathogens, however, they also accumulate organic matter that may inhibit extraction and qPCR quantification and therefore special measures are needed for method development.

**Figure 3:**
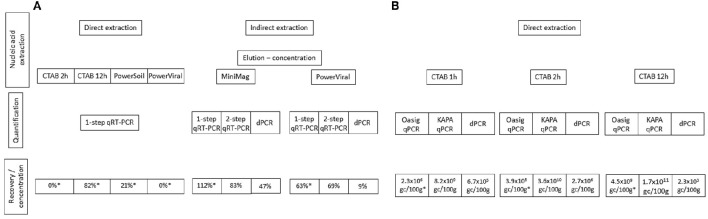
**Summary of the nucleic acid extraction and quantification methods used for the recovery of (A)** NoV and **(B)** *Vibrio* spp. Method efficiency is compared using recovery percentiles for norovirus and genome copy concentrations for *Vibrio* spp. qPCR results refer to 10 times diluted nucleic acid extracts.

Ideally nucleic acid extraction methods are suitable for the co-extraction of RNA and DNA originated from a wide range of microorganisms and viruses. Our results suggested that using CTAB and beadbeating for cell/viral lysis efficiently released bacterial and viral nucleic acids. The elution-concentration method using beef extract for elution and PEG6000 for precipitation described here and in Lewis and Metcalf (1988) has been shown to be suitable for the detection and quantification of enteric viruses in sediment (Figure 3).

Regardless of the nucleic acid extraction method used a ten times sample dilution was necessary prior to qPCR. The only exception was when the KAPA Robust qPCR mastermix was used to quantify *Vibrio* DNA. Sample dilution may not be needed if post-extract purification procedures are used, however these additional steps may enhance the degradation of viral and bacterial genomes. Nonetheless, the detection limit of qPCR-based approaches is generally very low (≤10 gc) and sample dilution prior to q(RT-)PCR would still allow the detection of 10–100 gc. Hence, even when sample dilution is necessary, the assay is far more sensitive than other available quantification methods e.g., culturing. Regardless of sample dilution prior to qPCR, quantification showed great variation depending on the approach used (Figure 3). The best recoveries were achieved using the RNA Ultrasense One-step qRT-PCR kit for viral RNA and the KAPA Robust qPCR mastermix for both bacterial DNA and viral cDNA. Interestingly, the dPCR approach we used throughout this study did not efficiently quantify the target DNA/cDNA sequences. As discussed in Section q(RT)-PCR vs. Digital PCR, this may have been a result of analytical error. Further, evaluation of dPCR is necessary in order to assess the usefulness of dPCR for the quantification of DNA in sediment samples.

## Conclusions and recommendations

Direct extraction of nucleic acids using the CTAB method has been shown to be suitable for the co-extraction of high abundance viral and bacterial nucleic acids, and further analysis of the sample would help to better understand the composition of microbial and viral communities and to estimate health risks.

For NoV, indirect extraction combined with RNA extraction using the MiniMag® System and with quantification using the RNA Ultrasense kit gave the best recoveries (Figure 3), however sample dilution prior to qRT-PCR was necessary for accurate quantification. Furthermore, the samples obtained via PEG precipitation may be used for the evaluation of viral infectivity for viruses that can be cultured *in vitro* (Lewis and Metcalf, 1988) and viral integrity assays. Therefore, we recommend the use of this elution—concentration protocol for different types of sediment, however, as suggested by others (Le Guyader et al., 2009; Mattison et al., 2009), extraction and quantification controls should be used to address assay performance.

The two-step RT-qPCR method described here also gave adequate results. Our results suggest that better adjusted conditions for the RT and/or the more advanced reverse transcriptase used during the separate RT step enabled full cDNA synthesis in the sample. In addition, two-step approaches may be a useful and cost-efficient tool when it is desirable to study different RNA viruses in a single sample. Currently, we do not recommend the application of 3D dPCR for nucleic acid quantification in environmental studies, however it may be a valuable tool for precise quantification when used along with q(RT-)PCR.

## Author contributions

KF and FH wrote the manuscript and undertook the experiments and data analysis. SM, JM, and DJ advised on experiment design. KF, FH, SM, JM, and DJ prepared manuscript for submission. All authors have approved the final version to be published.

## Funding

This work was funded by the Natural Environment Research Council (NERC) and the Food Standards Agency (FSA) under the Environmental Microbiology and Human Health (EMHH) Programme (NE/M010996/1).

### Conflict of interest statement

The authors declare that the research was conducted in the absence of any commercial or financial relationships that could be construed as a potential conflict of interest.
